# Gastric metastasis from renal cell carcinoma with submucosal invasion treated by surgical full-thickness resection: a case report

**DOI:** 10.1186/s40792-024-02036-z

**Published:** 2024-10-28

**Authors:** Nanako Magara, Naoto Takahashi, Yuta Takano, Kenji Takeshita, Naoki Toya, Fumiaki Yano, Ken Eto

**Affiliations:** 1 Department of Surgery, The Jikei University Kashiwa Hospital, 163-1 Kashiwashita, Kashiwa, Chiba 277-8567 Japan; 2 Department of Surgery, The Jikei University School of Medicine, 3-19-18 Nishi-shinbashi, Minato-ku, Tokyo 105-8471 Japan

**Keywords:** Renal cell carcinoma, Gastric metastasis, Gastric wedge resection, Full-thickness resection

## Abstract

**Background:**

Metastatic gastric tumors are rare and malignant melanoma, breast cancer, lung cancer, and esophageal cancer are common as primary lesions. On the other hand, renal cell carcinoma is easy to metastasize hematogenously to the whole body. However, metastasis to the stomach is rare and the detailed treatment of gastric metastasis is not mentioned. In this study, we report an uncommon case of gastric metastasis from renal cell carcinoma that underwent surgical full-thickness resection and reviewed the literature for treatment options.

**Case presentation:**

The patient was a female in her 60s and in January 2007, she underwent a transabdominal left nephrectomy for clear cell carcinoma of the left kidney. The pathological diagnosis was pT2N0M0 stage II. In October 2017, a total pancreatectomy with D2 dissection was performed for multiple pancreatic masses, in which the pathological diagnosis was pancreatic metastasis of renal cell cancer. In May 2019, an esophagogastroduodenoscopy for heartburn revealed redness and erosion in the greater curvature of the residual gastric body. The pathological diagnosis was gastric metastasis from renal cell carcinoma. No metastatic findings were observed and gastric wedge resection was performed. Pathological diagnosis of the resected specimen showed a 4-mm tumor, mainly within the mucosa and partly extended to the submucosal layer in 500 µm. The resected specimen had a clear resection margin.

**Conclusions:**

In this study, we report a case in which a full-thickness resection was performed for gastric metastasis 12 years after renal cancer surgery and 2 years after pancreatic metastasis surgery. The patient survived 4 years and 8 months after gastric wedge resection. Although gastric metastasis often takes the form of submucosal tumors, it is necessary to select full-thickness resection for R0 resection, even in small and flat lesions.

## Background

Metastatic gastric tumors are rare and malignant melanoma, breast cancer, esophageal cancer, and lung cancer are the most common primaries [1]. Gastric metastases of renal cell carcinoma are said to be sporadic at 0.65% [2]. On the other hand, it is estimated that 25–30% of renal cell carcinoma will have simultaneous transference and 40% will have metachronous transference [3], and metastatic organs are often lungs, liver, bones, and brain [4]. As for the metastasis of renal cell carcinoma to the digestive system, metastasis to the pancreas is relatively rare at 2.8% [5], while metastasis to the stomach is scarce at 0.2–0.7% [4].

According to the Clinical Practice Guideline for Renal Cancer, metastatic resection is expected to improve the survival rate if the patient has a good performance status, has a long disease-free period, and can be completely resected [6]. However, the detailed treatment of gastric metastasis is not mentioned. As a mode of tumor metastasis to the stomach, tumor cells in the blood are trapped in the gastric submucosal tissue with abundant blood flow and metastasize [7]. Meanwhile, Prudhomme et al. [8] reported 42 gastric metastases from renal cell carcinoma and seven of those lesions were treated endoscopically. However, no matter how small the tumor is, surgical full-thickness resection is more suitable than Endoscopic mucosal resection and Endoscopic submucosal dissection to accomplish R0 resection. In this study, we report a case of gastric metastasis from primary renal cell carcinoma that underwent surgical full-thickness resection, reviewed the literature on PubMed and the Japan Medical Abstracts Society for metastatic gastric carcinoma, and examined the treatment methods for the small metastasis in particular in lesions of 10 mm or less.

## Case presentation

The patient was a female in her 60s who underwent a transabdominal left nephrectomy for clear cell carcinoma of the left kidney in January 2007. The pathological diagnosis was pT2N0M0 stage II. In October 2017, a pylorus-preserving total pancreatectomy with D2 dissection was performed for multiple pancreatic masses, in which the pathological diagnosis was pancreatic metastasis of clear cell renal cell carcinoma with no lymph node metastasis.

**Fig. 1:**
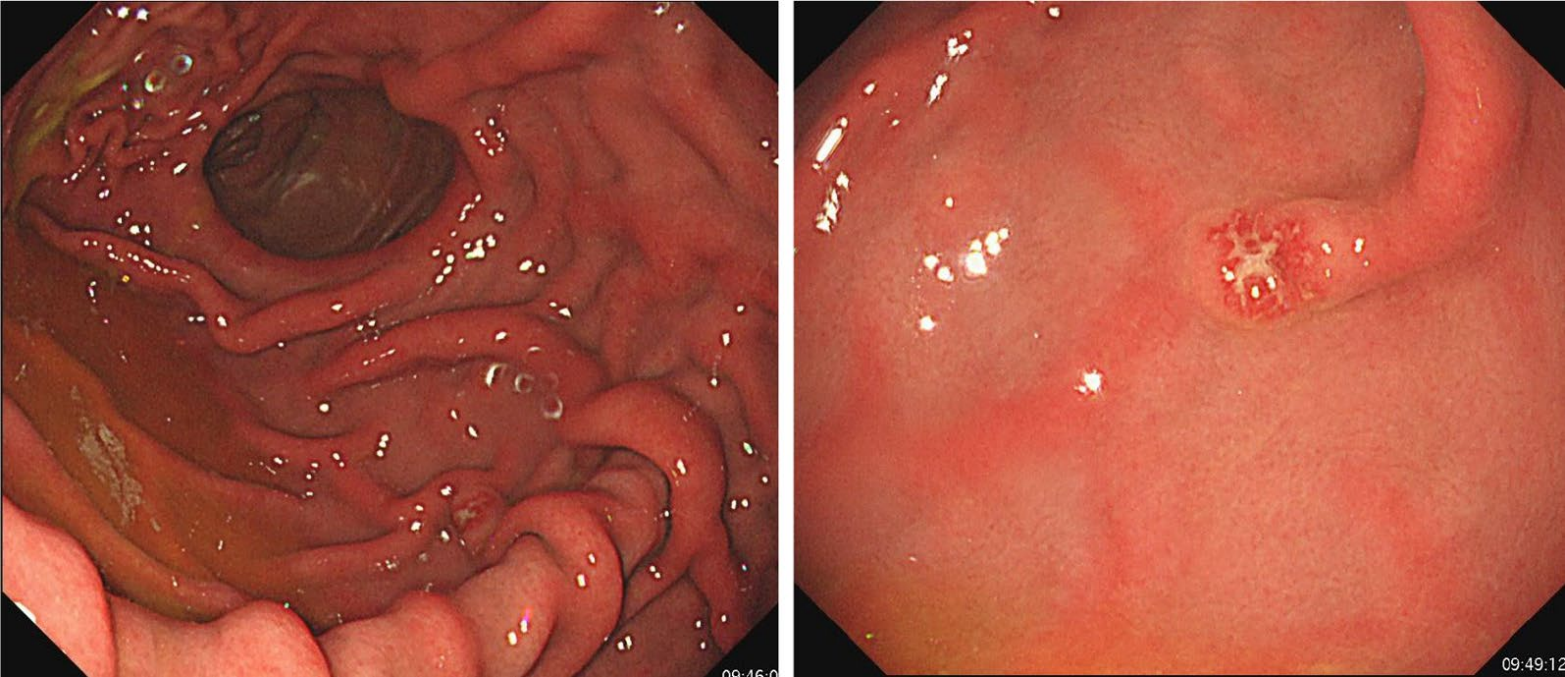
Esophagogastroduodenoscopy. A 5-mm redness erosion with an irregular depression in the center was observed on the greater curvature of the residual gastric body

**Fig. 2:**
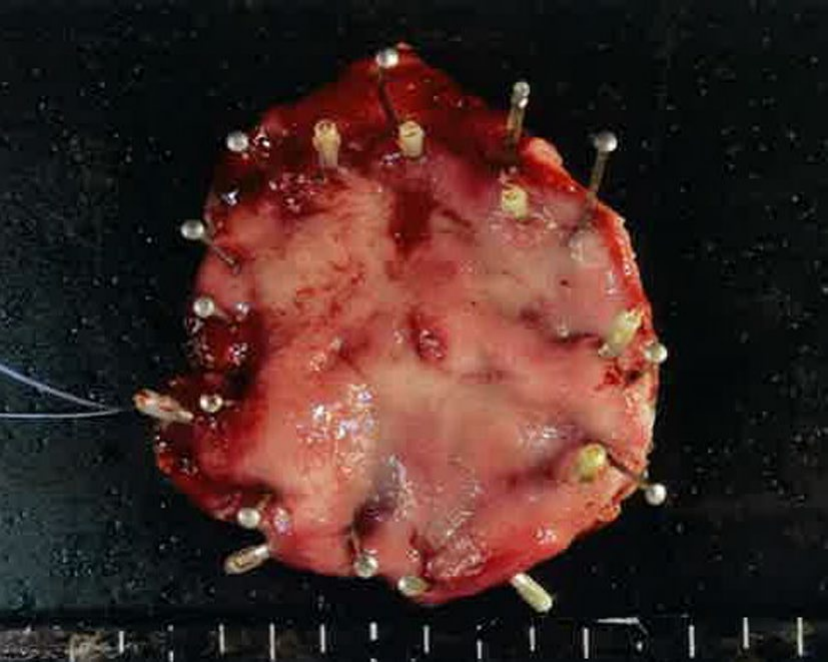
Pathological specimen. The lesion is resected with a margin of about 2 cm

**Fig. 3:**
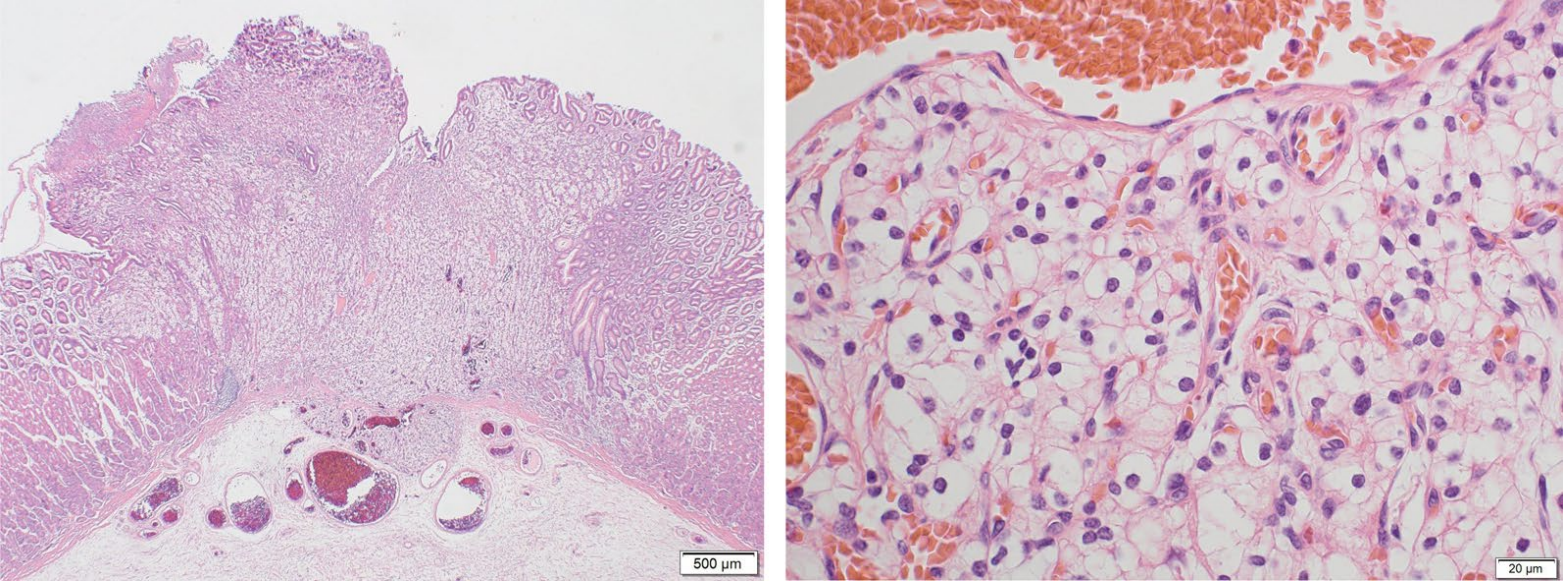
*Left side*: Pathological findings of hematoxylin and eosin staining ×20. Metastasis of 4 × 4 mm was observed in the mucosa and submucosal layer. The tumor in the submucosa was 500 µm in size, and the specimen had a venous invasion. *Right side* Pathological findings of hematoxylin and eosin staining ×20. Tumor cells with small circular nuclei and clear cytoplasm similar to those of the primary lesion developed in a honeycomb-like pattern

In May 2019, she visited our hospital for heartburn. Laboratory data reveal no abnormalities other than an increase in HbA1c (9.0%) associated with pancreatic diabetes. An esophagogastroduodenoscopy for heartburn revealed redness and erosion in the greater curvature of the residual gastric body (Fig. 1). The pathological diagnosis of the biopsy was gastric metastasis from renal cell carcinoma. No other metastases were found in enhanced computed tomography. After marking with endoscopic clips before surgery, gastric wedge resection by laparotomy with the support of intraoperative endoscopy was performed because severe adhesions were assumed after pancreaticoduodenal resection (Fig. 2). There were no postoperative complications and the patient was discharged on the 9th day. The tumor was 4 × 4 mm, existed mainly in the mucosa, and partly extended to the submucosal layer (Fig. 3-left side). The pathological diagnosis was metastatic clear cell renal cell carcinoma (Fig. 3-right side). The resected specimen had a clear resection margin. In April 2023, enhanced computed tomography pointed out multiple lung metastases. Chemotherapy was started in July 2023, and the patient is still alive in April 2024.

## Discussion

As a result of reviewing in PubMed, using the keywords “renal cell carcinoma” and “gastric metastasis”, and the Japan Medical Abstracts Society, using the keywords in Japanese “zingan” and “itenni”, only 94 cases and 109 lesions were selected [8–60], including our case. There are 22 lesions, of which the size of the tumor is 10 mm or more minor; 14 lesions, of which the size of the tumor is 11–20 mm; 17 lesions, of which the size of the tumor is 21–30 mm, and 28 lesions, of which the size of the tumor is more significant than 30 mm. The size of 28 lesions was not described.

Our case has a minor lesion of those 109 lesions and we examined the macroscopic morphology, the period from diagnosis of kidney cancer to the detection of gastric lesions, treatment methods, and prognosis regarding lesions, the size of which were 10 mm or more minor (Table 1). First, regarding macroscopic morphology, it has been reported that about 50% of metastatic gastric tumors show a submucosal tumor-like form, and about 40% show a primary gastric cancer-like form [1]. Table 1 shows polyp-like in 9 cases (40.9%), SMT-like in 7 cases (31.8%), and erosion in 6 cases (27.2%). This result was similar to Kim’s [51] report that the polyp-like form was the most common. Furthermore, gastric metastases of breast cancer often take the form of type 4 [61], and it should be noted that the phenotype in the stomach differs depending on the difference in the primary lesion. Next, we will consider treatment and pathological outcomes. Of the 22 patients examined in this study, 12 lesions were removed endoscopically, and only four were resected with full thickness by surgery. Pathological results of 22 cases from the resected tumors: 9 (40.9%) had submucosal invasion, 4 (18.2%) had mucosal cancer, and 9 (40.9%) had no description. The submucosal invasion was observed in 40.9% of gastric metastases as small as 10 mm or less. Our case has the most minor of all 109 lesions, but the most profound part lies in the submucosal area at 500 µm, meaning endoscopic submucosal dissection may not achieve R0. Some patients cannot endure surgery such as wedge resection; in those cases, endoscopic full-thickness resection can be helpful [62]. Therefore, we thought that it is essential to select full-thickness resection, for now, surgical full-thickness resection and, in the future, endoscopic full-thickness resection, to fulfill R0 resection when the gastric metastasis is resectable.

**Table 1:** Cases of gastric metastases from renal cell carcinoma (10 mm or smaller)

Case no.	Author	Year	Age/sex	Location	Size (mm)	Macroscopy	Depth of invasion	Therapy	Additional metastasis	Interval year	Outcome
1	Present case	2024	63/F	M	4	Erosion	SM	Wedge resection	Pancreas	12 years	56-month survival
2	Sogabe et al. [28]	2016	53/M	L	5	SMT like	SM	Chemotherapy	LN	2 years	14-month survival
3	Sogabe et al. [28]	2016	53/M	M	5	Erosion	SM	Chemotherapy	LN	2 years	14-month survival
4	Ishibashi et al. [33]	2017	63/M	M	5	SMT like	M	ESD	None	0 years	60-month survival
5	Koterazawa et al. [48]	2021	70/F	M	5	Erosion	ND	ESD	None	0 years	4-month survival
6	Hiroshige et al. [21]	2014	77/M	M	6	SMT like	ND	ESD	None	2 years	ND
7	Xu et al. [60]	2012	60/F	M	6	Polypoid	SM	Endoscopic treatment	None	0.4 years	Died 9-month AT
8	Xu et al. [60]	2012	60/F	M	6	Polypoid	SM	Endoscopic treatment	None	0.4 years	Died 9-month AT
9	Kim et al. [59]	2012	79/M	M	6	Erosion	SM	ESD	None	0 years	6-month survival
10	Chen et al. [49]	2022	65/M	U	4~6	SMT like	ND	Palliative ESD	Gallbladder, Pancreas, Soft tissue	5 years	8-month survival
11	Chen et al. [49]	2022	65/M	M	4~6	SMT like	ND	Chemotherapy	Gallbladder, Pancreas, Soft tissue	5 years	8-month survival
12	Yokota et al. [9]	2000	47/M	M	8	Polypoid	SM	EMR	Lung	6 years	ND
13	Ikari et al. [23]	2014	64/M	M	8	Polypoid	M	ESD	Pancreas	12 years	30-month survival
14	Otowa and Muto [63]	1992	61/F	U	10	Polypoid	ND	Total gastrectomy	None	0 years	Died 3-month AT
15	Sugamoto et al. [10]	2002	40/M	L	10	Erosion	ND	Chemotherapy, EMR	ND	4 years	3-month survival
16	Saidi et al. [58]	2007	ND	M	10	Polypoid	SM	Wedge resection	None	10 years	18-month survival
17	Harada et al. [17]	2011	65/M	U	10	Polypoid	M	EMR, Interferon	Bone	2 years	6-month survival
18	Arakawa et al. [35]	2018	80/F	M	10	SMT like	SM	Chemotherapy	Lung, Liver	0 years	ND
19	Kinoshita et al. [37]	2019	60/M	M	10	Erosion	M	LECS	Gallbladder	3 years	12-month survival
20	Weissman et al. [39]	2019	70/M	U	10	Polypoid	ND	ND	None	0 years	ND
21	Michigami et al. [56]	2023	81/F	M	10	Polypoid	ND	EMR	Pancreas	10 years	3-month survival
22	Su et al. [57]	2023	74/F	M	10	SMT like	ND	ESD	None	13 years	24-month survival

*ND* not described, *Interval year* the period from diagnosis of kidney cancer to the detection of gastric lesions, *U* upper third of the gastric body, *M *middle third of the gastric body, *L *lower third of the gastric body, *SMT* submucosal tumor, *EMR* endoscopic mucosal resecsion, *ESD* endoscopic submucosal dissection, *LECS* laparoscoppic and endoscopic cooperative surgery, *LN* lymph node, *AT* after therapy

## Conclusions

In this study, we report a case in which a full-thickness resection was performed for gastric metastasis 12 years after renal cancer surgery and two years after pancreatic metastasis surgery. The patient survived 4 years and 8 months after gastric wedge resection. Although gastric metastasis often takes the form of submucosal tumors, it is necessary to select full-thickness resection for R0 resection, even in small and flat lesions.

## Data Availability

All data generated or analyzed during this study are included in this published article.

## Abbreviations

ND: Not described
Interval year: The period from diagnosis of kidney cancer to the detection of gastric lesions
U: Upper third of the gastric body
M: Middle third of the gastric body
L: Lower third of the gastric body
SMT: Submucosal tumor
AGC: Advanced gastric cancer
EMR: Endoscopic mucosal resection
ESD: Endoscopic submucosal dissection
LECS: Laparoscopic and endoscopic cooperative surgery
LN: Lymph node
AT: After therapy
